# Comparison of different cardiovascular magnetic resonance sequences for native myocardial T1 mapping at 3T

**DOI:** 10.1186/s12968-016-0286-6

**Published:** 2016-10-04

**Authors:** Tiago Teixeira, Tarik Hafyane, Nikola Stikov, Cansu Akdeniz, Andreas Greiser, Matthias G. Friedrich

**Affiliations:** 1 Department of Cardiology, Philippa and Marvin Carsley Cardiovascular MR Centre; Montreal Heart Institute, Université de Montréal, Montréal, Canada; 2 Centro Hospitalar entre Douro e Vouga, Santa Maria da Feira, Portugal; 3 Neuroimaging Research Laboratory and École Polytechnique, Université de Montréal, Montreal, Canada; 4 Siemens Healthcare GmbH, Erlangen, Germany; 5 Departments of Medicine and Diagnostic Radiology, McGill University, Montreal, Canada

**Keywords:** Relaxometry, Native T1 mapping, SASHA, ShMOLLI, MOLLI

## Abstract

**Background:**

T1 mapping based on cardiovascular magnetic resonance (CMR) is a novel approach using the magnetic relaxation T1 time as a quantitative marker for myocardial tissue composition. Various T1 mapping sequences are being used, with different strengths and weaknesses. Data comparing different sequences head to head however are sparse.

**Methods:**

We compared three T1 mapping sequences, ShMOLLI, MOLLI and SASHA in phantoms and in a mid-ventricular slice of 40 healthy individuals (mean age 59 ± 7 years, 45 % male) with low (68 %) or moderate cardiovascular risk. We calculated global and segmental T1 in vivo through exponential curve fitting and subsequent parametric mapping. We also analyzed image quality and inter-observer reproducibility.

**Results:**

There was no association of T1 with cardiovascular risk groups. T1 however differed significantly depending on the sequence, with SASHA providing consistently higher mean values than ShMOLLI and MOLLI (1487 ± 36 ms vs. 1174 ± 37 ms and 1199 ± 28 ms, respectively; *p* < 0.001). This difference between sequences was much smaller in phantom measurements. In patients, segmental values were lower in the anterior wall for all sequences. Image quality, in general good for the steady-state-free-precession readouts in all sequences, was lower for SASHA parametric maps. On multivariate regression analysis, a longer T1 measured by MOLLI was correlated with lower ejection fraction and female gender. Inter-observer variability as assessed by intra-class correlation coefficients was excellent for all sequences (ShMOLLI: 0.995; MOLLI: 0.991; SASHA: 0.961; all *p* < 0.001).

**Conclusion:**

In a cross-sectional population with low to moderate cardiovascular risk, we observed a variation in T1 mapping results between inversion-recovery vs. saturation-recovery sequences in vivo, which were less evident in phantom images, despite a small interobserver variability. Thus, physiological factors, most likely related to B1 inhomogeneities, and tissue-specific properties, like magnetization transfer, that impact T1 values in vivo, render phantom validation insufficient, and have to be further investigated for a better understanding of the clinical utility of different T1 mapping approaches.

**Trial registration:**

“Canadian Alliance For Healthy Hearts and Minds” – ClinicalTrials.gov NCT02220582; registered August 18, 2014.

## Background

T1 mapping, which refers to the pixelwise quantification of the myocardial longitudinal relaxation time T1, is a rapidly emerging Cardiovascular Magnetic Resonance (CMR) technique which has demonstrated a strong potential to characterize abnormalities related to acute and chronic myocardial injury [20]. Global T1 measurements can be determined either by analyzing the intensity of a group of pixels in a parametric T1 map, but also by deriving an exponential T1 recovery curve based on ROIs from a number of steady-state free precession (SSFP) images with varied inversion times. [20]. Among the applied sequences, inversion-recovery techniques based on Look-Locker protocols (MOLLI, ShMOLLI) and saturation-recovery sequences (SASHA) have been used most frequently. Yet, more than 10 years after the first clinical study on myocardial T1 mapping [18, 19] and numerous reports on its utility for identifying different processes such as edema [8, 10] and diffuse fibrosis [2], the specific clinical utility of currently used protocols is not well understood. Specifically, there is a paucity of comparative data between these approaches beyond phantom experiments [13], especially in a suitable reference middle-aged population with low to moderate cardiovascular risk.

The aim of our study was to compare the ability of three major T1 mapping techniques, ShMOLLI, MOLLI 5(3)3 and SASHA, to determine the native relaxation T1 times in phantoms and in subjects from a population-wide cohort.

## Methods

### Phantom experiments

We performed experiments using two different Agarose and NaCl gel phantoms, here referred to as “HCMR” [16] (from the HCMR study: ClinicalTrials.gov NCT01915615) and “T1MES” [3], both composed of 3 × 3 array of plastic tubes filled with T1/T2 mixtures (T1 ~ 100–3000 ms and T2 ~ 50–200 ms), the tubes’ base resting on a resin layer. The two phantoms were scanned in the same MRI machine using identical imaging parameters as for the in-vivo T1 mapping experiments (see CMR acquisition parameters section below). T2 mapping was also performed using a T2-prepared TrueFISP sequence [12] at the same slice position as for the T1 mapping. A simulated heart rate of 60 bpm was used.

Reference T1 relaxation times were calculated offline based on five images collected using slice-selective IR with a turbo spin echo readout: Inversion times (TI) = 33, 100, 900, 2700, and 5000 ms, respectively; Temporal resolution (TR)/Echo time (TE) = 10s/12ms; Turbo factor = 7; 80 phase encoding steps with total image acquisition time of 372 s. Regions of interest were placed in each tube using an automated method and reference T1s were fitted per pixel and the mean T1 determined [1].

### Study population

Participants of the “Canadian Alliance For Healthy Hearts and Minds” prospective cohort (ClinicalTrials.gov NCT02220582) were offered to participate. The “Alliance” cohort is a joint Canadian effort to understand underlying risk factors for cardiac, vascular and cognitive dysfunction and will recruit more than 8,000 subjects from a multi-ethnic general population. Exclusion criteria for Alliance cohort are the generally accepted contraindications to MR (implantable devices, cochlear implants, cerebral aneurysm clips, severe claustrophobia), and renal dysfunction (current eGFR < 60 mL/min/1.73 m^2^).

Participants were recruited trough the Montreal Heart Institute (MHI) Biobank, consisting of patients of the MHI and their relatives. Between October 2014 and March 2015, 65 asymptomatic participants were recruited. After exclusion of participants with myocardial infarction (*n* = 6), valvular dysfunction (*n* = 6) or an implanted MR-incompatible device (*n* = 1), the presence of more than 3 risk factors (*n* = 5) or end-organ disease (*n* = 7), our study population consisted of 40 participants (mean age 58.7 ± 7.0; 45 % male).

History taking and assessment specifically included hypertension, *diabetes mellitus*, dyslipidemia, overweight (BMI > 30 kg/m^2^), smoking status, family history of premature cardiovascular events, and sedentary lifestyle. We used a qualitative cardiovascular risk score approach [17] to classify participants from “no added risk” (no risk factors) to “moderate added risk” (i.e. arterial hypertension with two or less cardiovascular risk factors, excluding diabetes mellitus; or diabetes mellitus, but without other risk factors).

### CMR acquisition parameters

All CMR scans were performed using a 3 Tesla clinical MRI system (Magnetom Skyra™, Siemens Healthcare, Erlangen, Germany), software platform Skyra VD13. We applied cine CMR sequences (steady state free precession) for ventricular function, mass and morphology [11], consistent with the standard scan protocol of the Alliance cohort, including three long axis views and a stack of short axis slices covering the entire heart, using the following parameters: Field of view (FOV) typically 320–350 mm; matrix 208x168; TE 1.43 ms; TR 39.12 ms; echo spacing 2.8 ms; slice thickness 8 mm with 2 mm gap; flip angle (FA) 49°; bandwidth (BW) 960Hz; 25 reconstructed phases per heartbeat.

We applied prototype sequences for T1 mapping (Siemens ASP #780B), in random order, all performed using 18-channel phased array body matrix coils (anterior and posterior). Patient-specific shimming and center frequency adjustments were performed before T1 mapping to generate images free from off-resonance artifacts. Regular parameters used were:

- ShMOLLI 5(1)1(1)1[23]: 7 SSFP readouts in 9 heartbeats; slice thickness 8 mm; FA 35°; TE 1.07 ms; TR 2.58 ms; sampling rate 100 %; acquisition matrix 192 × 144; FOV ~ 320–350 mm; 84 segments; phase partial Fourier 6/8; BW 900Hz; minimal TI 100 ms; TI increment, 80 ms; parallel imaging (GeneRalized Auto-calibrating Partially Parallel Acquisition, GRAPPA) with an acceleration factor of 2; imaging window 167 ms.
- MOLLI 5(3)3[14]: 8 SSFP readouts in 11 heartbeats; slice thickness 8 mm; FA 35°; TE 1.07; TR 2.58 ms; matrix 192×144; FOV ~ 320–350 mm; 72 segments; minimum TI 100 ms; TI increment 80 ms; GRAPPA acceleration factor 2; imaging window 136 ms.
- SASHA [5]: 11 SSFP readouts in 11 heartbeats; slice thickness 8 mm; composite saturation with six RF pulses, FA 70° (optimized BIR4-90); TE 1.07 ms; TR 2.58 ms; matrix 192 × 144; FOV ~ 320–350 mm; 84 segments; saturation times spaced uniformly over the RR interval with minimum saturation recovery time of 100 ms; phase partial Fourier 6/8; BW 900 Hz; GRAPPA acceleration factor 2, imaging window 167 ms.

All sequences provided raw SSFP readouts, parametric maps and error maps, and for MOLLI and SASHA motion-corrected SSFP readouts (Siemens in-built).

### T1 analysis

The analysis was performed in the CMR Core Lab at the Montreal Heart Institute, using certified postprocessing software (cvi^42^, Circle Cardiovascular imaging, Calgary, Canada), software version 5.1.2 (303), and according to published societal recommendations [11, 28].

#### Quantitative analysis

There are two ways to perform T1 measurements: 1) choose myocardial regions of interest (ROI) in SSFP readouts, either raw or after motion-correction (MOCO), and evaluate them in a devoted software that determines the T1 value by using exponential curve fitting, accounting for specific algorithms of each sequence used, which we will refer as ROI-based analysis; 2) use parametric maps, which already contain pixel-wise information on T1 values (in our case we could generate cvi^42^-derived maps along with the ones generated inline by the scanner), which are referred to as pixel-map based analysis. T1 quantification was performed first by manually delineating the endocardial and epicardial contours of the LV myocardium, on the parametric map provided inline by the scanner, using a color scale of 0 to 2000 ms. Contours were drawn ensuring a ROI within the myocardium and excluding neighboring pixels from blood or epicardial fat (pixel-map based analysis). These contours were then copied and pasted to the first motion-corrected SSFP image (when available), and forwarded to the remaining SSFP readouts (ROI-based analysis). The contours were adjusted for minimal respiratory motion induced position changes. T1 was automatically determined by the software, from exponentially fitted curves, after selection of sequence-specific algorithms (conditional fitting for ShMOLLI, Look-Locker correction for MOLLI and 2-parameter fitting for SASHA), and accepted, if R^2^ of the calculated T1 curve function was equal to or higher than 0.995.

We further generated parametric maps by the software (automatic in under 30 s), using the motion-corrected Siemens readouts, and used them to estimate global and segmental [4] T1 values, using the same contours as for the inline maps (pixel-map based analysis). Segments affected by artifacts, particularly susceptibility artifacts, were excluded from the analysis.

For MOLLI a correction factor of 1.03 was applied offline to the values obtained both on the ROI-based analysis and the cvi pixel-map based analysis, as recommended by Siemens for any third-party analysis software.

A second reader (CA) repeated the analysis in 10 healthy participants for assessment of inter-observer variability.

#### Qualitative analysis

For the three T1 mapping sequences, SSFP images and T1 maps were evaluated qualitatively in relation to image quality, using a three point scale: 1 – poor image quality (motion-correction artifacts, susceptibility artifacts involving all the myocardium or motion artifacts preventing border visualization on SSFP images; significant color heterogeneity or susceptibility artifacts involving all the myocardium on T1 maps); 2 – average image quality (small susceptibility artifacts on SSFP images or T1 maps); 3 – good image quality (absence of artifacts on SSFP images; signal homogeneity on T1 maps).

### Statistical analysis

Continuous variables are presented as mean ± standard deviation (SD). Comparison of normally distributed variables was performed by Student’s *t*-test, one-way analysis of variance (ANOVA) and repeated measures ANOVA (both with Bonferroni’s post-hoc test), as appropriate. Categorical variables were expressed as percentage and compared using the ***X*** ^2^ test, using a linear approach and Kendall’s *tau* for ordinal variables. Pearson’s correlation coefficient analysis was used to determine the stronger association between T1 measurements estimated by the ROI-based and pixel-map based approaches for in-vivo experiments, and for the correlation between sequences and reference T1 times in phantom experiments. Univariate predictors of T1 times were assessed with a multiple linear regression model. Fixed and proportional biases with 95 % limits of agreement were further assessed using Bland-Altman analysis. Inter-observer variability was assessed using intra-class correlation coefficients (ICC). All statistical analysis was performed using SPSS version 21 (SPSS, Inc., Chicago, IL). A two-sided *p* < 0.05 was considered statistically significant.

## Results

### Phantom experiments

**Fig. 1:**
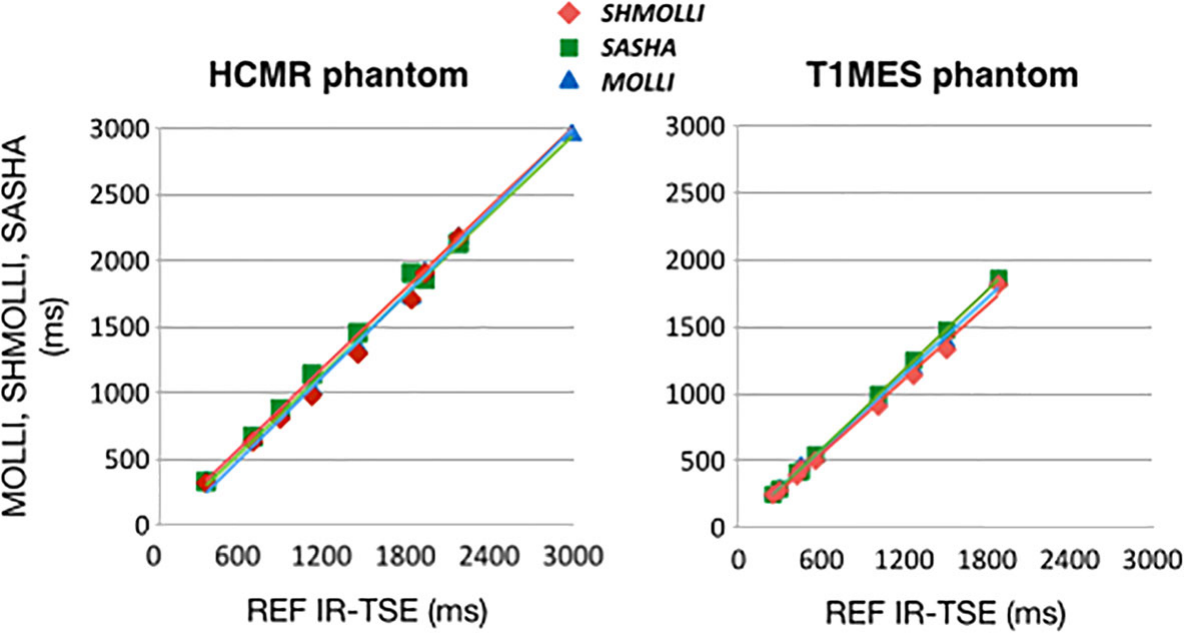
Native T1 for all sequences with different phantoms. The correlation of the measured T1 times and the predetermined T1 times was excellent ex-vivo, with an R^2^ = 0.99 on all sequences, for both phantoms

**Fig. 2:**
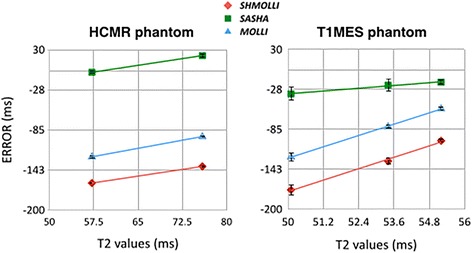
Impact of T2 on T1 measurements. MOLLI and ShMOLLI underestimated native T1 due to T2 effects, more prominently seen in shorter T2 times. SASHA was least affected by T2, as more obvious in images from the T1MES phantom (right panel)

**Fig. 3:**
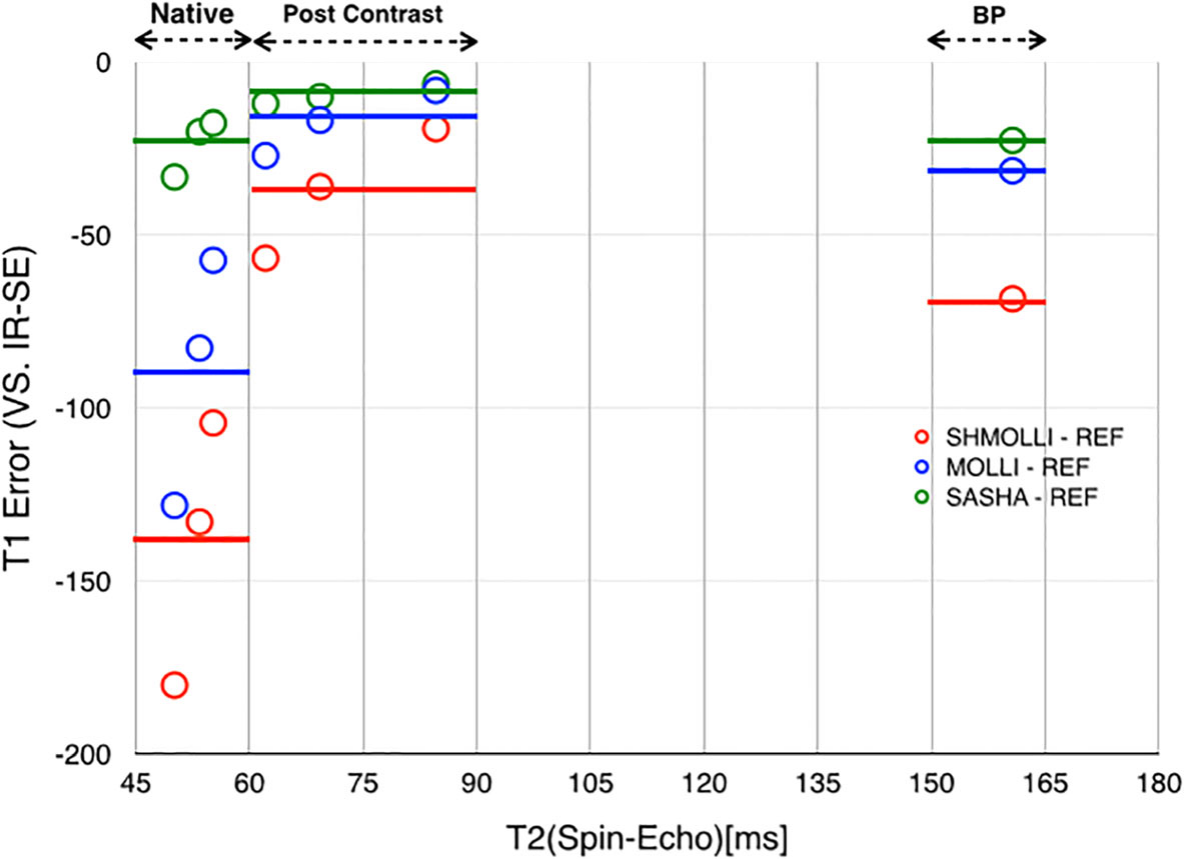
Error curves for the three sequences on T1MES. Absolute error in MOLLI, SHMOLLI and SASHA T1 values compared to gold standard inversion-recovery spin-echo (IR-SE) in native tissue-like phantom samples (T1s 1000–1500 ms, T2 50–55 ms), Post contrast tissue-like phantom samples (T1s 300–600 ms, T2 60–85 ms) and blood pool like phantom (T1 1880 ms, T2 160 ms). Continuous lines represent averages

In the in-vitro experiments, all sequences showed excellent correlation with the reference T1 times, as shown in Fig. 1 (for “HCMR” phantom: ShMOLLI y = 1.02x – 99.75, SASHA y = 0.99x – 5.41, MOLLI y = 0.98x – 29.26; for T1MES phantom: SASHA y = 0.99x – 8.25, MOLLI y = 0.95x – 6.62, ShMOLLI y = 0.92x – 4.51). The inversion-recovery techniques, particularly ShMOLLI, showed slight underestimation of T1 (~ 150 ms) (Fig. 2), while SASHA was the sequence least affected by increasing T2 values, presenting the smallest slope in the error curve, fact more evident when using T1MES, a more recent phantom that better accounts for this factor (T1MES curve has three different T2 values, ranging from 50 to 55 ms, closer to in-vivo reality when compared to HCMR’s curve, with only two, 57 and 75 ms). We further calculated the absolute error of the estimated T1 by the three sequences, against the gold standard inversion recovery spin-echo, for T1MES (Fig. 3). We verified that SASHA shows the best agreement (−23.73 ± 8.38), and both MOLLI and ShMOLLI show greater underestimation (respectively, −89.47 ± 35.93 and −139.23 ± 38.27), for the different T1 and T2 values.

**Fig. 4:**
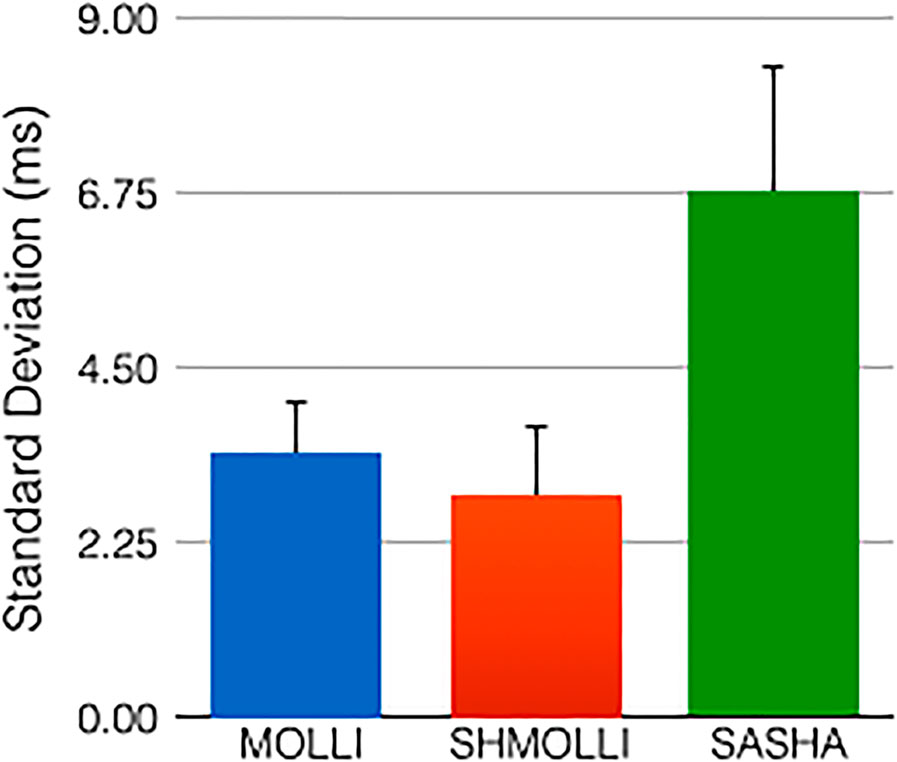
Comparison of the observed precision of sequences on phantom experiments. SASHA showed a higher standard deviation between measurements when compared to both ShMOLLI and MOLLI (p < 0.05)

SASHA showed the lowest precision, with higher standard deviation between measurements as compared to both ShMOLLI and MOLLI, *p* < 0.05 (Fig. 4).

## Participant studies

Recorded conditions at inclusion of the subjects in the database were: absent (62.5 %), negative ischemic evaluation (22.5 %), rhythm disorder (10 %) or other (5 %). Demographic characteristics, clinical and CMR results are presented in Table 1. We analyzed the different added-risk groups: normal volunteers (without any risk factors); low added-risk group (presence of 1–2 risk factors, without known hypertension); and hypertensives (with or without at most two other risk factors on top of hypertension). The hypertensive group, mostly constituted of moderate added-risk patients, had higher prevalence of dyslipidemia (*p* < 0.01), while the low added-risk group had a higher prevalence of a family history of premature CV disease (*p* < 0.01). The latter had also higher prevalence of risk factors (*p* < 0.001), but in 78 % of the cases only one risk factor was present. No other differences were found between groups, namely in routine MR parameters or T1 values within sequences.

**Table 1:** Demographic characteristics and CMR results in participants

Variables	Healthy	Low added risk	Moderate added risk	Sig
	(*n* = 9)	(*n* = 18)	(*n* = 13)	(*p* value)
Age (years)	58.4 ± 11.1	59.2 ± 5.4	58.2 ± 5.7	0.93
Male gender (%)	33.3	44.4	53.8	0.64
Diabetes M. (%)	0	0	15.4	0.11
Dyslipidemia (%)	0	11.1	61.5	< .01
Obesity (%)	0	27.8	23.1	0.22
Current smoker (%)	0	11.1	0	0.57
Sedentary (%)	0	0	0	–
FH CVD (%)	0	72.2	38.5	< .01
Hypertension (%)	0	0	100	< .001
SBP (mmHg)	129.3 ± 21.4	134.3 ± 16.8	136.9 ± 20.2	0.66
DBP (mmHg)	74.8 ± 9.1	81.8 ± 9.2	79.3 ± 15.8	0.35
RF (0/1/2/3)	9/0/0/0	0/14/4/0	0/2/4/7	< .001
LVEDV (ml)	123.8 ± 26.4	121.9 ± 35.1	129.5 ± 29.6	0.80
LVESV (ml)	45.8 ± 13.4	39.3 ± 16.1	41.1 ± 12.8	0.55
EF (%)	63.1 ± 6.0	68.3 ± 6.2	68.4 ± 5.1	0.07
LVM (g)	99.9 ± 23.3	112.6 ± 31.3	127.8 ± 35.1	0.11
Native T1				
ShMOLLI (ms)	1177.7 ± 28.6	1166.8 ± 24.3	1178.7 ± 51.7	0.70
MOLLI 5(3)3 (ms)	1207.9 ± 18.2	1197.0 ± 20.9	1206.8 ± 39.0	0.51
SASHA (ms)	1486.0 ± 24.1	1479.8 ± 29.6	1496.9 ± 48.9	0.44

*FH* family history, *CVD* cardiovascular disease, *SBP* systolic blood pressure, *DBP* diastolic blood pressure, *RF* risk factors, *LVEDV* left-ventricular end-diastolic volume, *LVESV* left-ventricular end-systolic volume, *EF* ejection fraction, *LVM* left-ventricular mass, *Sig* significance

### Comparison of T1 measurements from different sequences in vivo

Resulting T1 values differed between sequences. Using a ROI-based analysis, SASHA resulted in values almost 300 ms higher than those acquired by the other two sequences, ShMOLLI and MOLLI (ShMOLLI 1173.6 ± 37.0; MOLLI 1199.2 ± 27.5; SASHA: 1486.8 ± 36.0; *p* < 0.001). The post-hoc analysis, including only 32 participants due to the exclusion of 8 ShMOLLI curves with a *R* ^2^ < 0.995, shows significant differences between all sequences (Table 2). Heart rates did not differ between sequences.

**Table 2:** Comparison of mid-ventricular native T1 values as derived from different T1 mapping sequences

Sequences	ShMOLLI	MOLLI 5(3)3	SASHA	*p* value
T1 values (ms)	1173.6 ± 37.0	1200.3 ± 29.1	1492.7 ± 31.6	< 0.001
Heart rate (bpm)	65.7 ± 11.8	65.7 ± 11.8	66.5 ± 12.1	0.21

*ms* Milliseconds, *bpm* beats per minute

### Comparison of T1 analysis methods

**Fig. 5:**
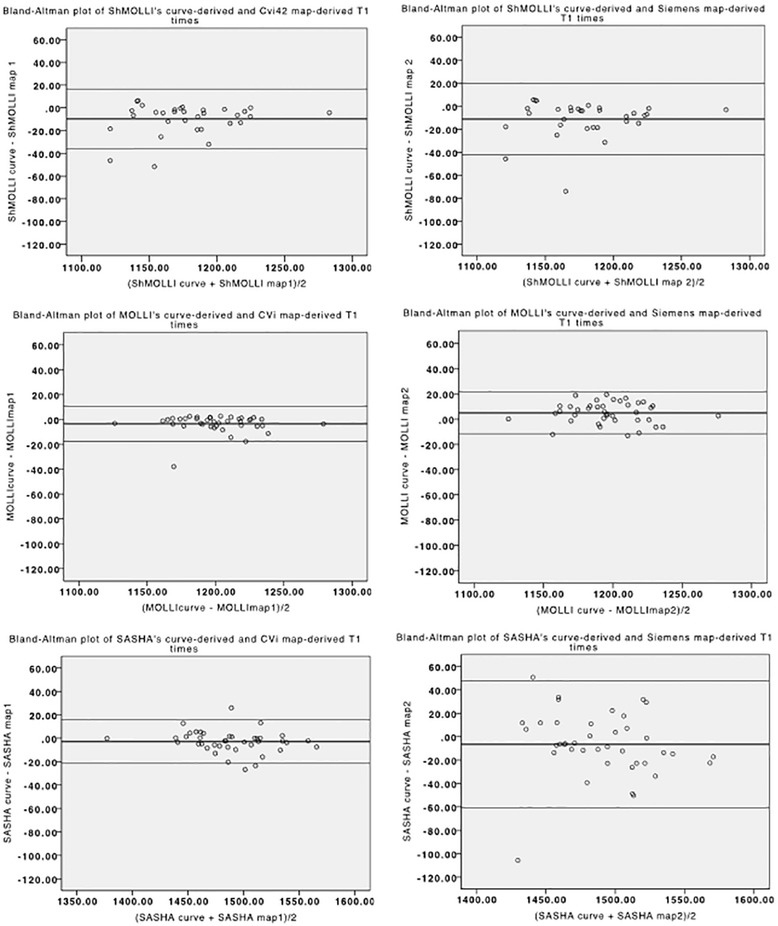
Bland-Altman plots of the difference between native T1 measurements using ROI-based and pixel-map based analysis, either generated by offline analysis (left) and inline/scanner-generated analysis (right), for the three sequences. ShMOLLI’s and MOLLI’s graphs are similar, but SASHA’s measurements show a lower dispersion when using the offline maps

We further calculated the T1 times using both sets of parametric maps (pixel-map based analysis), the ones originated inline from the scanner and the ones derived from the analysis software. Using the ROI-based analysis as standard, both sets of maps perform fairly equally, particularly for ShMOLLI (r: 0.90 vs 0.93; dif −11.23 vs 9.75) and MOLLI (r: 0.95 vs 0.97; dif 4.9 vs −3.4), but with a stronger correlation with cvi^42^ parametric maps when using SASHA (r: 0.74 vs 0.97; dif −6.51 vs −2.90) (Fig. 5).

**Fig. 6:**
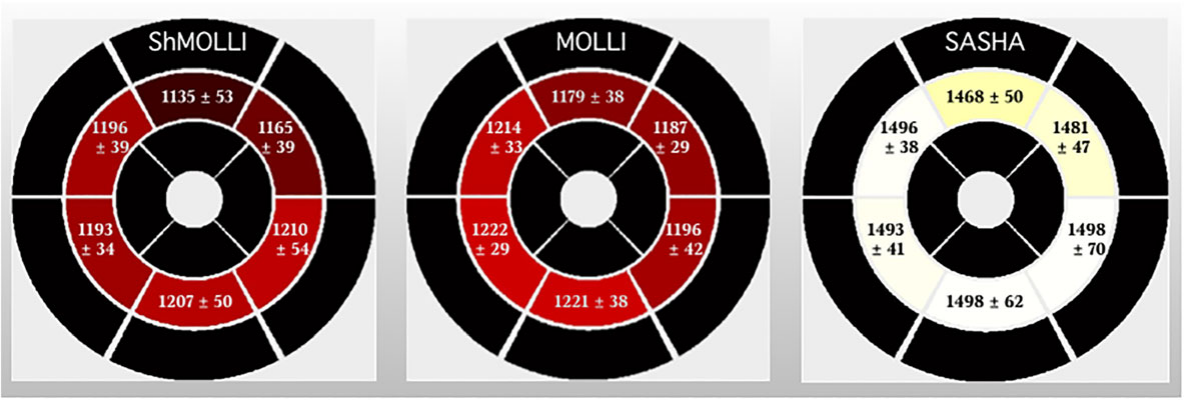
Segmental analysis of native T1 times using parametric maps. Bright yellow colors represent higher T1. T1 is consistently lower in the anterior segments than in the inferior and infero-lateral segments (p < 0.01). Such an inhomogeneity is less evident in T1 results acquired by SASHA

Segmental analysis was conducted in the parametric maps, (as generated by cvi^42^), the results shown in Fig. 6. The anterior segment had consistently lower T1 times in all the sequences, with higher values in the inferior/infero-lateral segments (*p* < 0.01). Regarding the segmental analysis, we excluded 0.7 % of the segments of SASHA and MOLLI sequences, due to susceptibility artifacts. For ShMOLLI, 17.7 % of the segments were excluded, but only 1 % of these were due to susceptibility artifacts (not statistically different from other sequences); the remaining 16.7 % corresponded to the aforementioned 8 ShMOLLI cases without acceptable fitting curves, related to heart rate mis-registration (a technical problem in our scanner).

### Image quality

**Fig. 7:**
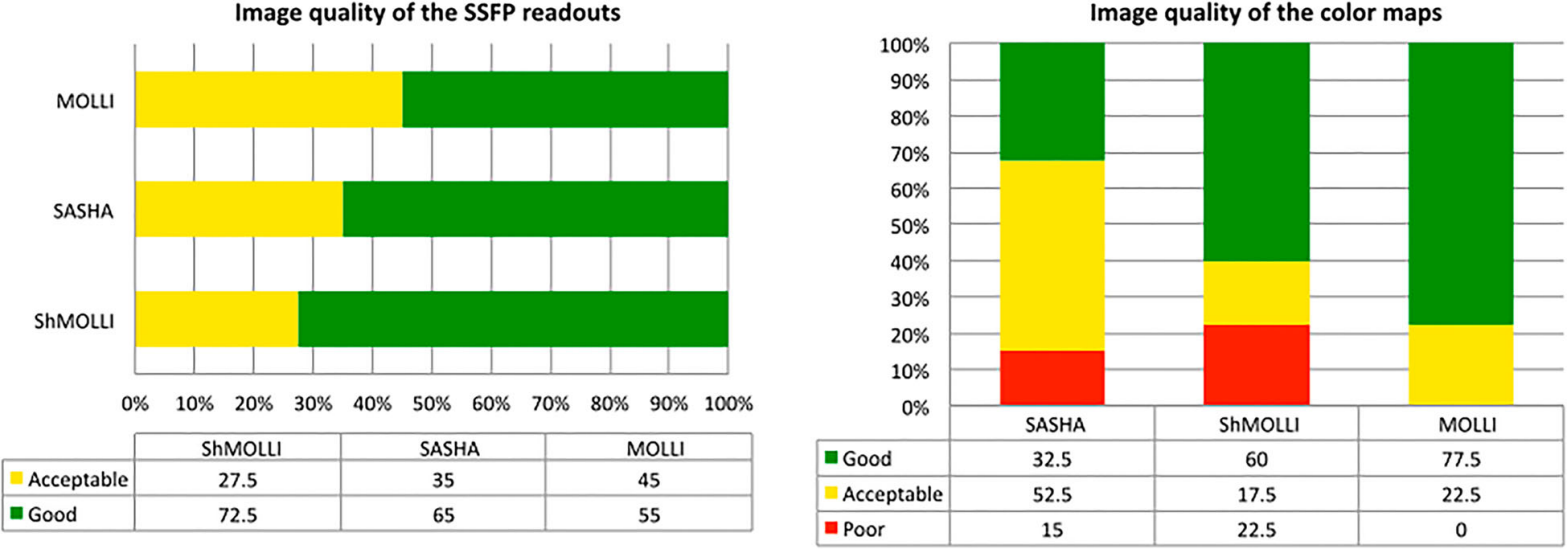
Evaluation of image quality between sequences. On the left the grey steady-state free precession (SSFP) readouts, and on the right the parametric color maps. Values are expressed as percentage. There was good overall image quality of SSFP images, with some susceptibility artifacts, affecting mostly MOLLI. Image quality was poorer on SASHA parametric maps, with about 2/3 showing less-than-good quality

**Fig. 8:**
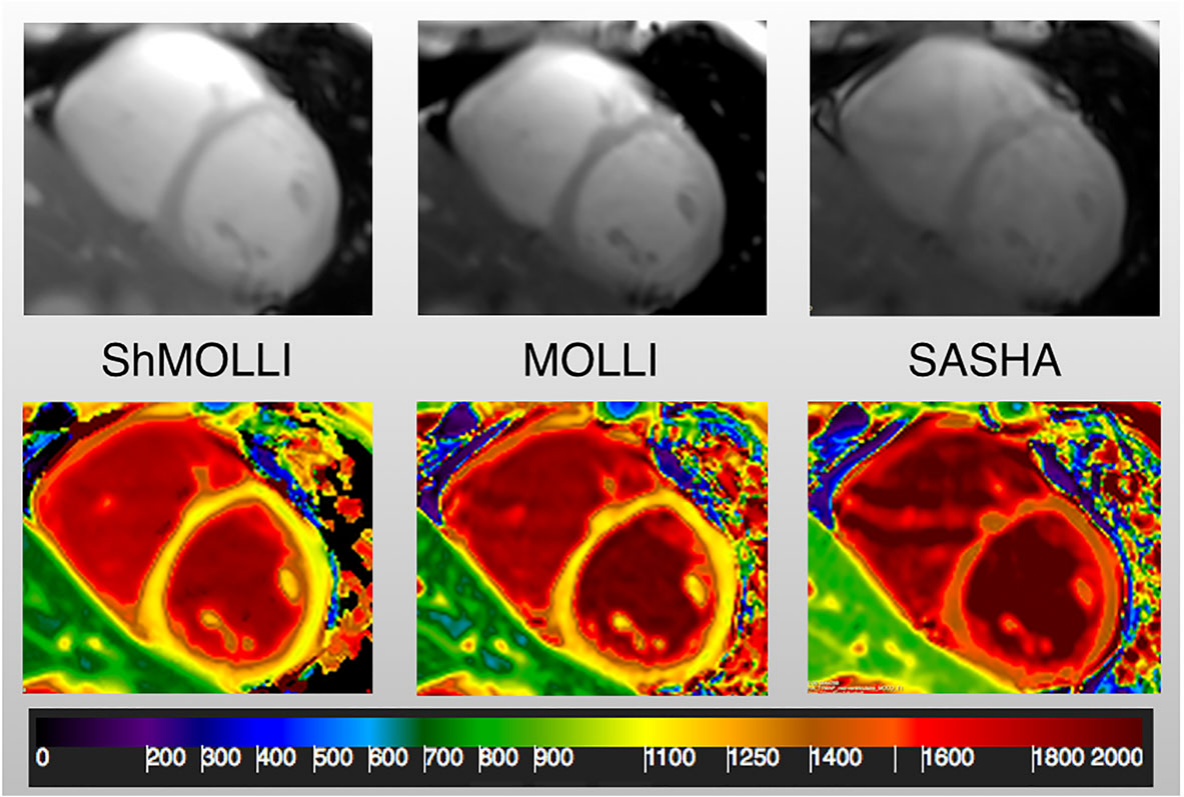
Example of representative SSFP images (top) and color maps (bottom) for each sequence, from left to right ShMOLLI, MOLLI and SASHA. Notice the susceptibility artefact present in all sequences (infero-lateral wall), in the SSFP images. The scale was chosen to keep the same interval (200 ms) in the range of each sequence (900–1100 ms for ShMOLLI and MOLLI; 1400–1600 ms for SASHA)

Fig. 7 shows an overview of the observed image quality. All the SSFP readouts had acceptable or good quality, with susceptibility artifacts progressively less identified in MOLLI, SASHA and ShMOLLI images (*p* < 0.001). Parametric maps had a lower quality with the SASHA approach when compared with ShMOLLI (*p* < 0.01) and MOLLI (*p* < 0.05), showing a significant percentage of heterogeneous maps, seldom present in other sequences’ maps. Representative examples of the SSFP readouts and parametric maps are shown in Fig. 8.

### Determinants of T1 values

**Fig. 9:**
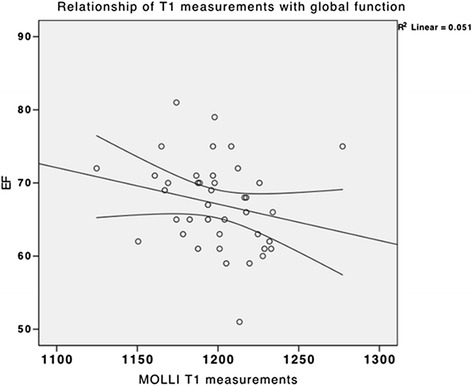
Scatter plot showing a weak negative correlation between ejection fraction and native T1 times estimated by MOLLI. The multivariate model included age, gender, ejection fraction and presence of hypertension and obesity

A multivariate analysis including age, gender, ejection fraction and presence of hypertension and obesity, showed significant relationship with the model (*r* = 0.54) in MOLLI only, with a negative correlation with ejection fraction (Fig. 9) and a positive correlation with female gender (both *p* < 0.05).

### Inter-reader variability

The intraclass correlation coefficients were excellent between both readers for the three sequences, when T1 was evaluated using the ROI-based analysis (ShMOLLI: 0.995; MOLLI: 0.991; SASHA: 0.961; all *p* < 0.001). The coefficients for T1 evaluation using the parametric maps, were still excellent overall, albeit lower (0.888) in the anterolateral segment of MOLLI images and the anterior (0.841) and infero-septal (0.886) segments in the SASHA images.

## Discussion

Our results indicate that, despite excellent inter-observer variability and good in-vitro correlation of various T1 mapping sequences, results vary in vivo between saturation-recovery sequences and inversion-recovery approaches. Furthermore, saturation-recovery sequences may provide less homogenous parametric maps (Fig. 5).

Native T1 of tissue in vivo can be estimated from signal intensities in images with varying inversion or saturation times through calculating an exponential T1 recovery curve [20]. The original Look-Locker technique was modified to a single-breath-hold technique with steady-state free precession (SSFP) readouts, known as Modified Look-Locker Inversion recovery (MOLLI) sequences [19]. This approach was further modified by Piechnik et al. with a Shortened adaptation with conditional curve fitting/ShMOLLI [23], allowing shorter breath-holds and less heart rate susceptibility. Modified MOLLI versions with fewer off-resonance artifacts were then necessary [14]. However, all these techniques still require a correction factor to account for underestimation of T1 due to magnetization attenuation [9]. Furthermore, T2 affects the results and, while T1 and T2 changes typically are correlated, the T1 acquired by MOLLI techniques may not represent a “true” T1. Chow et al. added a protocol based on images with varying saturation times, called SAturation recovery Single-sHot Acquisition (SASHA) sequence [5], which showed good accuracy [13]. No correction factor is necessary and there is no significant impact of tissue T2, but the curve fitting assumes a fully saturated tissue environment, which may not be present. Hybrid sequences are also available, that integrate some of the advantages of saturation and inversion-recovery [31].

All these techniques have different issues and while a consensus group is working on standardizing terminology and techniques [20], problems remain regarding the standardized use of techniques and the lack of accepted reference values. While some diseases such as cardiac amyloidosis or acute myocardial infarction show a strong increase in native T1, more subtle differences are obtained in conditions with less myocardial damage such as valvular heart disease with LV hypertrophy [27]. Thus, even small differences between calculated results limit the clinical utility of T1 mapping, if sequences and evaluation procedures are not standardized.

Reported reference values for the different techniques were acquired in studies with variable sample sizes and for different field strengths [7, 24, 30]. MOLLI sequences result in reference values (mean ± 2*SD) of 900 and 1050 ms at 1.5 T, while at 3 T myocardial T1 was found to be between 1100 and 1250 ms [21, 22]. Our results, obtained in a cross-sectional, middle-aged population at a low to moderate risk, are similar. Furthermore, we did not find a significant correlation with the cardiovascular risk profile, which is consistent with two recent studies, one by Dabir et al. ﻿[7], where a low-risk population had similar T1 values as normal volunteers, and one by Sado et al. with a reported overlap of T1 times of normal subjects and patients with arterial hypertension [27].

In our study, T1 as estimated by ShMOLLI and MOLLI was significantly lower than estimated by SASHA. While there are no data on reference values for T1 measurements based on SASHA at 3T, the observed difference is consistent with a previous review by Kellman and Hansen [13], who found a difference of about 200 ms between inversion and saturation-based methods, when determined at 1.5T. We observed a similar difference in the phantom experiments, which was amplified on the in-vivo measurements. In their review, Kellman and Hansen discuss a variety of factors that may affect T1 mapping accuracy, such as patient and tissue characteristics, protocol parameters, scanner and sequence adjustments, and fit models. In our sample the heart rate during SASHA acquisition was not different from the other sequences, being low (66.5 ± 12.1) and very close to the heart rate used in the phantom experiments, where there was not a wider difference in T1 estimation between sequences. Other tissue characteristics may further impact results relative to the phantom models, mostly in inversion-recovery techniques, leading to lower T1 values. In particular, magnetization transfer (MT) may be responsible for about 15 % of the assumed underestimation of T1 times with MOLLI, while this confounding effect is reduced with SASHA, a sequence that is less dependent on T2, at least when using a 3-parameter fit [25]. Magnetization transfer may therefore have been one of the major contributors to the inter-sequence differences. In our case, both scanner and sequence parameters were kept unchanged throughout all experiments. The SASHA fitting routine assumes perfect saturation (90° flip angle), but B1+ inhomogeneities and variations in the transmit gain could produce deviations from 90° and result in a T1 bias. Although using different sequences, Stikov et al. [29] reported similar findings in the brain, suggesting improper B1 correction as the reason for poor agreement between sequences in vivo (with variable flip angles), but not in phantoms. Importantly, a variable flip angle approach for SASHA has been reported recently as a means of reducing systematic errors [6].

The lower precision of SASHA, i.e. a higher variability of results, is a known disadvantage [26]. We also observed a marked heterogeneity of T1 compared with the other sequences. Different curve fit algorithms or sampling schemes may reduce this problem [15]. Our results did not seem to be related to artifacts in the SSFP readouts. For example, even though SASHA had fewer susceptibility artifacts in the SSFP images, the resulting T1 maps were of lower quality than maps based on MOLLI.

We used a ROI-based analysis for T1 measurements because the ROI-based approach allows for corrections of motion-correction imperfections or problems with cardiac phase registration. The pixel-map based approach is however a fair alternative, as shown in our study. The maps, as main overall advantage, enable a qualitative interpretation, priceless on clinical evaluation. That type of interpretation is quite difficult when using the SSFP readouts of the ROI-analysis, due to the aggressive windowing needed to define the myocardium on images with different T1s (shades of gray). We found the qualitative interpretation to be more frequently achieved with the inversion-recovery sequences, due to the referred homogeneity issues we encountered with SASHA’s maps. The maps further allow the analysis of myocardial segments. We confirmed the findings of other groups, with lower T1 at the anterior segments and higher values in the inferior/infero-lateral segments [30]. While a physiologic phenomenon cannot be completely excluded, it more likely represents an artifact.

Our study had several limitations. The sample is small and heterogeneous, and suffered further from missing heart rate data from the scanner. Even though we excluded those values from the analysis, the pixel-map based analysis from the Siemens inline generated maps, could have allowed for T1 estimation when we experienced this error (average T1 on 7 out of 8 cases: 1176 ± 18 ms). Nevertheless, the impact of cardiovascular risk on T1 has to be studied in larger samples, to confirm the significant differences of measured T1 between sequences. This is a field at constant development, and any analysis may risk to become outdated by the time of reporting. Marked improvements have, in fact, been implemented on some of these sequences, specifically a variable flip angle approach for SASHA that may have led to smaller observed differences between sequences and reduced homogeneity issues with that sequence. We only acquired a single myocardial slice and variations may be more or less pronounced in basal and apical slices. Yet, the observed differences and our conclusions on clinical utility of T1 mapping sequences remain valid.

## Conclusion

While performing T1 mapping in phantoms and in a cross-sectional population with low to moderate cardiovascular risk, we observed a small interobserver variability of T1 measurements. The MOLLI sequence showed the smallest overall variability and SASHA the best accuracy. Most importantly, measured T1 varied between inversion-recovery vs. saturation-recovery sequences in vivo, much more than in phantom images. Thus, physicians applying or interpreting the technique should be aware that phantom validation does not directly translate to in vivo application, because physiological factors, most likely related to B1 inhomogeneities, the position of the heart in the coil reception field, T2 sensitivity, magnetization transfer effects and physiologic variations impact on T1 values (whether between different methods like IR vs SR, or within a single method), and have therefore to be further investigated for a better understanding of the clinical utility of different T1 mapping approaches.

## Abbreviations

ANOVA: Analysis of variance
BMI: Body mass index
bpm: Beats per minute
BW: Bandwidth
CMR: Cardiovascular magnetic resonance
CVD: Cardiovascular disease
DBP: Diastolic blood pressure
EF: Ejection fraction
FA: Flip angle
FH: Family history
FOV: Field of view
GFR: Glomerular filtration rate
GRAPPA: Generalized auto-calibrating partially parallel acquisition
ICC: Intra-class correlation coefficients
LV: Left ventricle
LVEDV: Left-ventricular end-diastolic volume
LVESV: Left-ventricular end-systolic volume
LVM: Left-ventricular mass
MHI: Montreal Heart Institute
MOLLI: Modified look-locker inversion recovery
MRI: Magnetic resonance imaging
ms: Milliseconds
MT: Magnetization transfer
RF: Radiofrequency
RF: Risk factors
ROI: Region of interest
SASHA: SAturation recovery Single-sHot Acquisition
SBP: Systolic blood pressure
SD: Standard deviation
ShMOLLI: Shortened modified look-locker inversion recovery
Sig: Significance
SSFP: Steady-state free precession
TE: Echo time
TI: Inversion time
TR: Temporal resolution
